# *N*-Alkyl Derivatives of Deoxynojirimycin (DNJ) as Antiviral Agents: Overview and Update

**DOI:** 10.3390/molecules31030399

**Published:** 2026-01-23

**Authors:** Paola Checconi, Domenico Iacopetta, Alessia Catalano, Jessica Ceramella, Maria Maddalena Cavalluzzi, Annaluisa Mariconda, Stefania Marsico, Stefano Aquaro, Pasquale Longo, Maria Stefania Sinicropi, Giovanni Lentini

**Affiliations:** 1 Department for the Promotion of Human Sciences and Quality of Life, San Raffaele University, Via di Val Cannuta 247, 00166 Rome, Italy; paola.checconi@uniroma5.it; 2 Laboratory of Microbiology, IRCCS San Raffaele Roma, Via di Val Cannuta 247, 00166 Rome, Italy; 3 Department of Pharmacy, Health and Nutritional Sciences, University of Calabria, 87036 Arcavacata di Rende, Italy; domenico.iacopetta@unical.it (D.I.); jessica.ceramella@unical.it (J.C.); stefania.marsico@unical.it (S.M.); s.sinicropi@unical.it (M.S.S.); 4 Department of Pharmacy-Drug Sciences, University of Bari “Aldo Moro”, Via Orabona, 4, 70126 Bari, Italy; mariamaddalena.cavalluzzi@uniba.it (M.M.C.); giovanni.lentini@uniba.it (G.L.); 5 Department of Basic and Applied Sciences, University of Basilicata, Via dell’Ateneo Lucano, 10, 85100 Potenza, Italy; annaluisa.mariconda@unibas.it; 6 Department of Life, Health and Environmental Sciences, University of L’Aquila, Piazzale Salvatore Tommasi, 1, Blocco 11, Coppito, 67010 L’Aquila, Italy; stefano.aquaro@univaq.it; 7 Department of Chemistry and Biology “A. Zambelli”, University of Salerno, Via Giovanni Paolo II, 132, 84084 Fisciano, Italy; plongo@unisa.it

**Keywords:** deoxynojirimycin, UV-4, UV-4B, *N*-9′-methoxynonyl-1-deoxynojirimycin, antivirals

## Abstract

*N*-Alkyl deoxynojirimycin-derived drugs, belonging to the class of iminosugars, are well-known for their α-glucosidase inhibitory activity. *N*-Butyl-deoxynojirimycin (*N*-butyl-DNJ; NB-DNJ; also known as miglustat or UV-1) has been developed for the treatment of type 1 Gaucher disease and Niemann–Pick disease type C as Zavesca^®^. Furthermore, it behaves as a host-targeted glucomimetic that inhibits endoplasmic reticulum α-glucosidase I and II (GluI and GluII, respectively) enzymes, resulting in improper glycosylation and misfolding of viral glycoproteins; thus, it is a potential antiviral agent. It is studied against a broad range of viruses in vitro and in vivo; however, its utility as antiviral has not been fully explored. Other *N*-alkylated congeners of DNJ are in preclinical and clinical studies for diverse viral infections. The iminosugar *N*-9′-methoxynonyl-1-deoxynojirimycin (MON-DNJ or UV-4) is probably the most studied and potent inhibitor of α-Glu I and α-Glu II in clinical trials. It is often studied in the form of its hydrochloride salt (UV-4B) and has broad-spectrum activity against diverse viruses, including dengue and influenza. In clinical trials, it was found to be safe at all doses tested up to 1000 mg. In this paper, an overview on *N*-alkyl derivatives of DNJ is reported, focusing on their antiviral activity. The literature search was carried out by means of three literature databases, i.e., PubMed/MEDLINE, Google Scholar, and Scopus, screened using different keywords. A brief history of the discovery of their usefulness as antivirals is given, as well as the most recent studies on new compounds belonging to this class. Since different names are often used for the same compound, we tried to dissipate confusion and bring some order to this jumble of names. Specifically, in the tables, all the diverse names used to identify each compound, were reported.

## 1. Introduction

Deoxynojirimycin (DNJ, moranoline, duvoglustat) is an α-glucosidase (EC 3.2.1.20) inhibitor as well as a pharmacological chaperone of acid α-glucosidase [1]. It is an alkaloid contained in *Morus alba* L., a plant with high nutritional value [2]. Several biological activities, including antihyperglycemic, lipid-lowering, antitumor, anti-inflammatory, and antiviral, have been reported for this compound. Focusing on the latter, the first evidence of antiviral activity of DNJ emerged in the 1980s from studies on influenza virus [3], followed by vesicular stomatitis virus (VSV) [4] and Sindbis virus [3,5]. The study by Datema et al. (1987) [6] evidenced that, in intact cells, DNJ exerts its action on both GluI and GluII, with higher activity on GluII. The latter is a heterodimeric enzyme bearing a catalytic α-subunit of the GH31 family and an accessory β-subunit that is necessary for the full catalytic activity and localisation of the heterodimer in the endoplasmic reticulum (ER). An in-depth study on the key molecular differences between GluI and GluII has been recently reported in detail in a review by Oo et al. (2025) [7]. The inhibition of glucosidases for the antiviral activity was further investigated in retroviruses, such as Moloney murine leukemia virus [8], human immunodeficiency virus (HIV) [9,10], and other viruses [2,11].

However, most studies regarding DNJ are focused on its derivatives and congeners, rather than on DNJ as it is [12,13]. Several derivatives of DNJ have been studied for their diverse biological activities, and research in this field continues [14]. Miglitol (Glyset^®^) received FDA approval for type 2 diabetes in 1996, while miglustat (Zavesca^®^) addresses lysosomal disorders, specifically Gaucher and Niemann–Pick diseases (from 2002 in the EU and from 2003 in the USA) [15]. These drugs are currently under study against other diseases, including melanoma [16], cardiac fibrosis [17], mitochondrial dysfunction [18], cystic fibrosis [19], periodontitis [20], and to ameliorate radioresistance in patients with cancer [21]. Interestingly, several *N*-alkylated congeners have demonstrated good antiviral properties, but their use in therapy is still under investigation. Some *N*-alkylated-DNJ congeners are included in the host-directed antivirals (HDA) category, which represents a promising avenue for broad-spectrum treatment, also with reduced risk of resistance development [22]. These compounds inhibit host α-glucosidases, which are pivotal for the glycoprotein folding endoplasmic reticulum quality control (ERQC) [23]. The external α-1,2-linked glucose is cleaved by endoplasmic reticulum (ER) GluI, and the inner α-1,3-linked glucoses are removed by ER GluII during *N*-linked glycan processing [24]. The impaired glucose cleavage results in misfolded proteins, which are either degraded by ER-associated degradation or secreted with altered properties. Thus, the treatment of virus-infected cells with iminosugars disrupts the proper virion morphogenesis [23]. The antiviral activity exerted by these compounds has been demonstrated against various viruses [25,26], including both enveloped DNA viruses, as HBV and positive-strand RNA viruses, namely flaviviruses, such as bovine viral diarrhea virus (BVDV), Dengue virus (DENV), and West Nile virus (WNV) [27], human hepatitis C virus (HCV) [28], and coronaviruses [29], namely Severe Acute Respiratory Syndrome Coronavirus 1 (SARS-CoV-1) [30] and Severe Acute Respiratory Syndrome Coronavirus 2 (SARS-CoV-2) [31]. In this review, we summarize the *N*-alkyl derivatives of DNJ studied as antivirals. In the first part, we focused on the most common ones, also reporting the history of this class of compounds, while in the second part, some studies on the latest *N*-alkyl derivatives of DNJ are highlighted. The search was conducted using PubMed/MEDLINE, Google Scholar, and Scopus. The search criteria considered the occurrence of the association of different keywords: “deoxynojirimycin”, “*N*-alkyl-deoxynojirimycin” or “*N*-alkyl-DNJ” “-derivatives”, “*N*-9′-methoxynonyl-1-deoxynojirimycin”, “miglustat”, “*N*-nonyl-DNJ”, “*N*-[*N*-(4-azido-2-nitrophenyl)-6-aminohexyl]-DNJ” and the acronyms “NN-DNJ”, “MON-DNJ”, “NAP-DNJ”, “ToP-DNJ”, “UV-4”, “UV-4B”, “UV-5” in association with “antivirals”, “preclinical studies”, “clinical trials”, and “clinical studies”. The most relevant studies reporting the antiviral activity published in the English language were selected for preparing the review.

## 2. A Brief History of the Discovery of the Antiviral Activity of *N*-Alkyl Analogs of DNJ

**Figure 1:**
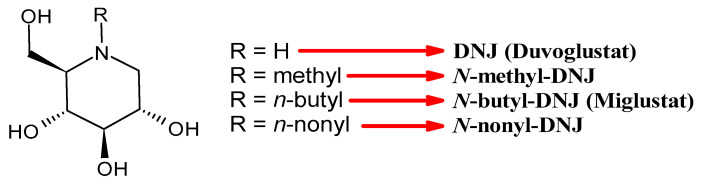
Structure of deoxynojirimycin (DNJ) and its first studied *N*-alkyl derivatives.

The first compounds belonging to the class of *N*-alkyl derivatives of DNJ are reported in Figure 1. These compounds act on the biosynthesis of *N*-linked-oligosaccharides. It begins with the transfer of the oligosaccharide Glc_3_Man_9_GlcNAc_2_ from a dolichol carrier to acceptor asparagine residues in the nascent protein. Trimming to high mannose type oligosaccharides Man_9-5_GlcNAc_2_ is initiated cotranslationally in the ER, starting with the stepwise removal of the three glucose residues (1–3). GluI cleaves off the terminal α-l,2-linked glucose, and GluII the two inner α-l,3-linked glucoses. The oligosaccharides are further processed within the ER and Golgi apparatus, where the trimming of mannose residues and conversion to complex type oligosaccharides occur by the addition of *N*-acetyl glucosamine, galactose, fucose, and sialic acid residues [32]. In 1983, Romero et al. [33] studied the antiviral activity of DNJ and derivatives in fowl plague virus-infected chicken-embryo cells, i.e., an avian influenza A virus. This study revealed that the *N*-methylation of DNJ made it a more specific inhibitor of glucosidase activity than DNJ (which also inhibited the dolichol-oligosaccharide formation step) (Figure 1). In contrast to DNJ, it was found that *N*-methyl-DNJ mainly inhibited the action of GluI [6,34]. A successive study by Karpas et al. [9] reported a method to score in parallel both the degree of antiviral activity and the effect on cell division of iminosugars, specifically *N*-alkyl derivatives of DNJ, including *N*-methyl-DNJ and *N*-butyl-DNJ (Figure 1). This evidence showed that, in intact cells, they significantly inhibited HIV replication, with *N*-methyl-DNJ reducing the yield of infectious HIV by an order of four logarithms, whereas *N*-butyl-DNJ reduced the virus titer by more than five logarithmic orders at noncytotoxic concentrations. Tan et al. (1991) [35] studied some other alkyl derivatives of DNJ, specifically *N*-methyl-, *N*-butyl-, *N*-pentyl-, *N*-isobutyl, *N*-isopentyl-, and *N*-benzyl-DNJ. It was confirmed that the *N*-alkylated derivatives were able to inhibit GluI more than GluII. The inhibition of glucosidase activity exerted by *N*-alkylated derivatives of DNJ was less easily reversed than that by DNJ, and the effect was more pronounced for *N*-methyl-DNJ. Branching of the alkyl group of DNJ derivatives decreased the inhibitory potency. Block et al. (1994) [36] demonstrated that *N*-butyl-DNJ was also able to suppress the secretion of hepatitis B virus (HBV) particles and caused intracellular retention of HBV DNA. In 1995, Fischer et al. suggested that the mechanism of action of *N*-butyl-DNJ as an inhibitor of HIV replication was the impairment of viral entry at the level of post-CD4 binding, as the interaction of gp120-CD4 is preserved, and probably due to an effect on other viral envelope components [37]. In 1998, Block et al. [38] studied the *N*-nonyl-DNJ, a 9-carbon alkyl derivative of DNJ (Figure 1). Although it contained a long branching, it reduced the viremia in woodchucks chronically infected with woodchuck hepatitis virus (WHV) (which is a naturally occurring pathogen of woodchucks, belonging to the same family of human HBV, hepadnaviridae) in a dose-dependent manner, and it was 100–200 times more potent than *N*-butyl-DNJ in inhibiting HBV in cell-based assays [39]. Then, Zitmann et al. (1999) [40] investigated both *N*-butyl- and *N*-nonyl-DNJ against BVDV, a tissue culture surrogate of HCV, demonstrating that in MDBK cells, both α-glucosidase inhibitors prevented the formation and secretion of infectious BVDV. The authors also found that *N*-nonyl-DNJ exhibited a longer retention in the liver in vivo than *N*-butyl-DNJ. Mehta et al. (2002) [41] analyzed fourteen *N*-alkyl derivatives of DNJ by using a virus secretion assay for HBV and a single-step growth assay for BVDV. Thanks to structure–activity relationship studies, Mehta et al. (2002) [42] suggested that side chains of at least eight or nine carbon atoms were important for achieving maximal antiviral activity. Some differences were found with the introduction of an oxygen atom. Generally, the so-called “rule of nines” establishes that a side chain length of 8–9 carbons of *N*-alkyl-DNJ derivatives, and a Log*P* of approximately 2.8–3.0, seems to be the optimal compromise between antiviral efficacy and acceptable cytotoxicity [14]. Sayce et al. (2016) [43] confirmed that both *N*-butyl- and *N*-nonyl-DNJ repressed DENV production via inhibition of ER α-glucosidases and not glycolipid processing enzymes. Determination of antiviral efficacy of *N*-butyl-DNJ and *N*-nonyl-DNJ in primary human monocyte-derived macrophages (MDMΦs) infected with DENV led to an EC_50_ of 10.6 µM and 1.5 µM, respectively. However, in the study by Warfield et al. (2017) [44], *N*-butyl-DNJ showed in vitro activity against two filoviruses, Ebola and Marburg viruses (EBOV and MARV, respectively), while showing minimal effects on the survival times of EBOV- and MARV-infected mice receiving the compound. However, *N*-butyl-DNJ was found to be unable to reach sufficient antiviral serum concentration in vivo due to tolerability issues. Durantel et al. (2001) [45] investigated the mechanism of antiviral activity of *N*-butyl- and *N*-nonyl-DNJ against BVDV, showing that the long-alkyl-chain induced an envelope glycoprotein homodimer accumulation in the ER. Wu et al. (2002) [46] studied the *N*-nonyl-DNJ against Japanese encephalitis virus (JEV) and dengue virus serotype 2 (DEN-2) infections, demonstrating once again the antiviral activity of this compound against flaviviruses. Several other DNJ derivatives were evaluated in vitro for activity against flaviviruses, specifically BVDV [27,47], DENV [48], and HCV [49], and were extensively summarized by DeWald et al. (2020) [50]. It was demonstrated that the α-glucosidase inhibition underlies the antiviral effect of iminosugars in DENV infection in MDMΦs [43,51]. Kiappes et al. (2018) [52] reported that iminosugars may inhibit GluII at lower concentrations and GluI at higher concentrations; however, the inhibition of GluII alone leads to measurable anti-flavivirus activity. With the arrival of the “tsunami”, the COVID-19 pandemic, and the post-COVID disease [53], these compounds have returned to the forefront [54]. Brun et al. (2025) [31] reported a study on SARS-CoV-2 variants focused on diverse antivirals, among which some include *N*-alkyl derivatives of DNJ, specifically, *N*-butyl-DNJ (NB-DNJ), *N*-nonyl-DNJ (NN-DNJ), *N*-9′-methoxynonyl-1-DNJ (MON-DNJ), and *N*-(*N*-4-azido-2-nitrophenyl)-6-aminohexyl-1-deoxynojirimycin (NAP-DNJ). The *N*-9′-methoxynonyl derivative of DNJ demonstrated antiviral activity against SARS-CoV-2 wild type as well as alpha, beta, gamma, delta, and omicron variants. This compound also showed activity against betacoronavirus HCoV OC43. Two DNJ derivatives, specifically *N*-butyl-DNJ (UV-1) and UV-4, have been advanced to phase I or phase I/II clinical trials for the antiviral therapy [50].

## 3. An Overview of *N*-Alkyl Derivatives of DNJ

In this section, the most studied *N*-alkyl derivatives of DNJ are summarized (Table 1). The first naturally occurring *N*-alkyl derivative of DNJ, *N*-methyl-DNJ, was isolated from the leaves and roots of *Morus* spp. (mulberry trees) [55]. However, this compound, although useful for structure–activity relationships, was overcome by other later-studied derivatives. Thus, *N*-methyl DNJ will not be detailed below. For the other *N*-alkyl derivatives of DNJ, the most characterizing antiviral activities have been taken into consideration. Half maximal inhibitory concentration (IC_50_) and half maximal effective concentration (EC_50_) are reported, calculated against diverse viruses, as detailed in the table. Half maximum cell cytotoxicity (CC_50_) was evaluated against mammalian and human cell lines, including Vero, human lung adenocarcinoma (Calu-3), human hepatocarcinoma (Huh-7), Madin–Darby bovine kidney (MDBK), and human hepatoma (HepG2 2.2.15), as detailed in the table.

### 3.1. Most Common N-Alkyl Derivatives of DNJ as Antivirals

In this subsection, we report an overview of the most common *N*-alkyl derivatives of DNJ (UV-1–UV-5) with their antiviral activities (Table 1).

#### 3.1.1. *N*-Butyldeoxynojirimycin (*N*-Butyl-DNJ or NB-DNJ or UV-1 or AT2221, Miglustat)

*N*-Butyldeoxynojirimycin (*N*-butyl-DNJ or NB-DNJ or UV-1 or AT2221, miglustat) is the four-carbon N-linked side chain DNJ derivative, which was approved in 2003 for Gaucher’s Disease [66] and in the European Union (EU), Canada, and Japan for the treatment of Niemann–Pick disease type C [67,68,69] as Zavesca^®^. The reversible inhibition of ceramide glucosyltransferase is the basis for its type I Gaucher’s disease approval, while the ability of this compound to cross the blood–brain barrier enables the treatment of lysosomal storage diseases with neurological manifestations, thus justifying its use in the management of neurogenerative Niemann–Pick disease type C. Several recent studies have been carried out on this drug for the treatment of Pompe Disease, by examining the combination with a recombinant human acid alpha-glucosidase (ATB200, cipaglucosidase alfa, Pombiliti™, Princeton, NJ, USA) (NCT02675465) [70,71]. A phase III double-blind randomized clinical trial (NCT03729362) to study the efficacy and safety of intravenous ATB200 co-administered with oral AT2221 (*N*-butyl-DNJ) in adults with Late Onset Pompe Disease has been recently completed [72]. The clinical trial NCT04327973 is still open and is addressed to patients with Infantile-Onset Pompe Disease. Several studies are related to the antiviral activity of *N*-butyl-DNJ. The effects on HIV-1 infection of this compound were examined [73], along with the combinations with nucleoside analogs (dideoxyinosine, dideoxycytidine, and azidothymidine) [74,75]. Miller et al. (2012) [56] demonstrated that *N*-butyl-DNJ can reduce production of infectious DENV in an MDMΦ model using blood from dengue-naïve human donors. Moreover, *N*-butyl-DNJ was studied against SARS-CoV-2 [76]. Rajasekharan et al. (2021) [58] studied miglustat against SARS-CoV-2 and found it was active, suggesting it as a repositional drug for the treatment of COVID-19. El Khoury et al. (2024) [77] reported that *N*-butyl-DNJ exerts inhibitory effects on SARS-CoV-2 S1 secretion, ACE2 trafficking, and subsequently reduces S1/ACE2 interaction, thus providing a promising therapeutic potential for COVID-19.

#### 3.1.2. *N*-Nonyl-DNJ (NN-DNJ or UV-2)

*N*-Nonyl-DNJ (NN-DNJ or UV-2) bears a nine-carbon linear alkyl side chain and showed a remarkable improvement in potency with respect to *N*-butyl-DNJ, being 100–200 times more potent in inhibiting hepatitis B virus (HBV) in cell-based assays [39]. *N*-Nonyl-DNJ also exhibited remarkable inhibitory activity against three influenza A viruses of the H3N2 and H1N1 subtypes in a strain hemagglutinin-dependent manner, indicating its potential to inhibit virus replication and transmission, which was 10-fold more potent than *N*-butyl-DNJ [61]. It was also studied as an antiviral against filoviruses, EBOV and MARV, in vitro using a yield-plaque assay and in vivo animal models of EBOV and MARV, showing interesting results [44]. *N*-Nonyl-DNJ can reduce the production of infectious DENV in the MDMΦ model using blood from dengue-naïve human donors, as reported by Miller et al. (2012) [56]. The study by Sayce et al. (2016) [43] confirmed that the class of iminosugars blocks DENV via inhibition of ER α-glucosidases, and not by inhibiting glycolipid processing. *N*-Nonyl-DNJ was also found to inhibit human papillomavirus (HPV) E5 channel activity in vitro [59]. This compound is also under study for Gaucher disease, acting as a potent N370S β-GCase inhibitor [78], and in vitro in colon cancer cell lines [79].

#### 3.1.3. *N*-7-Oxadecyl-DNJ (UV-3) (SP116)

*N*-7-Oxadecyl-DNJ was named as UV-3 and studied against DENV, EBOV, and MARV [14,44,57]. However, it showed generally lower activity than other derivatives. It was less cytotoxic than *N*-nonyl-DNJ [14].

#### 3.1.4. *N*-9′-Methoxynonyl-1-deoxynojirimycin (MON-DNJ or UV-4)

*N*-9′-Methoxynonyl-1-deoxynojirimycin (MON-DNJ or UV-4) is probably the most studied and potent inhibitor of α-Glu I and α-Glu II. Perry et al. (2013) [57] studied UV-4 for controlling DENV infection and disease in a mouse model. The antiviral activity of UV-4 was demonstrated against dengue virus serotype 2 (DENV-2) in multiple mouse models. The authors found that administration of UV-4 reduced mortality, as well as viremia and viral RNA in key tissues, and cytokine storm, suggesting its use as a DENV-specific antiviral. In phase I human clinical trials, MON-DNJ was active against EBOV and much more tolerable [44]. Stavale et al. (2015) [80] studied the in vivo therapeutic protection against influenza A (H1N1) oseltamivir-sensitive and resistant viruses exerted by UV-4 in mice, finding that it was highly efficacious via oral gavage against both viruses even if treatment was initiated as late as 48–72 h after infection, with a minimal effective dose of 80–100 mg/kg when administered orally three times daily. Sayce et al. (2021) [81] demonstrated that pathogen-induced inflammation is attenuated by the MON-DNJ via modulation of the unfolded protein response. Clarke et al. (2021) [76] demonstrated that UV-4 prevented SARS-CoV-2-induced cell death and reduced viral replication after 24 h of treatment; however, the antiviral effect was lost after 48 h.

#### 3.1.5. *N*-9′-Methoxynonyl-1-deoxynojirimycin Hydrochloride (UV-4B)

UV-4B is the hydrochloride salt of UV-4, i.e., *N*-9′-methoxynonyl-1-deoxynojirimycin hydrochloride [62]. It showed broad-spectrum antiviral activity, and it has recently been tested in humans against DENV infection. In phase I studies, UV-4B has been demonstrated to be well-tolerated in humans with no severe side effects observed after a single dosage of 1000 mg, and pharmacokinetic data indicated a low interindividual variability and good linearity over a wide range of dosages (NCT02061358). In the study by Warfield et al. (2017) [44], UV-4B was tested in a proof-of-concept, nonhuman primate model (rhesus monkeys) of EBOV infection. It failed to yield any survival benefit to macaques infected with EBOV-Makona, despite showing definitive antiviral activity against EBOV in vitro. UV-4B has completed phase I studies in healthy subjects in order to determine the safety, tolerability, and pharmacokinetics of this drug (NCT02061358) and the safety and pharmacokinetics of multiple ascending doses to healthy subjects (NCT02696291) [82,83,84]. In these trials, UV-4B proved to be safe at all doses tested up to 1000 mg after administration as an oral solution formulation and showed efficacy against measles and respiratory syncytial virus, suggesting the further development of UV-4B for antiviral activity [63,81,82]. Its activity also extended to another betacoronavirus, HCoV OC43 [17]. Warfield et al. (2020) [85] demonstrated that a single dose of this drug prevented the death of mice infected with lethal doses of influenza or dengue viruses, even when treatment was begun as late as 48 h post-infection. In 2022, Franco et al. [63] showed that UV-4B demonstrated strong anti-SARS-CoV-2 activity in A549-ACE2 cells (EC_50_ = 4 µM). Franco et al. (2021) [86] also demonstrated the antiviral potential of UV-4B in combination with interferon-alpha against DENV. UV-4B in combination with EIDD-1931, the main circulating metabolite of molnupiravir, has been suggested as a promising therapeutic strategy against SARS-CoV-2, as it inhibits replication of multiple SARS-CoV-2 strains in clinically relevant human cell lines (ACE2-A549 and Caco-2) [87].

#### 3.1.6. *N*-[*N*-(4-Azido-2-nitrophenyl)-6-aminohexyl]-1-deoxynojirimycin (NAP-DNJ or UV-5)

*N*-[*N*-(4-Azido-2-nitrophenyl)-6-aminohexyl]-1-deoxynojirimycin (NAP-DNJ or UV-5) is a six-carbon linear side chain containing derivative, which was initially synthesized by Rawlings et al. (2009) [65] as a potential photoaffinity probe for ER GluI and ER GluII. It was then shown to be a highly potent GluI inhibitor (IC_50_ = 17 nM) in an in vitro assay and proved to be equally effective at inhibiting cellular ER glucosidases in a free oligosaccharide analysis. Caputo et al. (2016) [88] found that it demonstrates high potency in the inhibition of mammalian ER GluII. NAP-DNJ also showed activity against EBOV but was less cytotoxic (CC_50_ = 350 μM) than NN-DNJ, but more than MON-DNJ. This compound, along with MON-DNJ, was selected to be further studied against EBOV and MARV in vivo, but in vivo efficacy in rodents and nonhuman primates was not demonstrated, as in this case [44]. The synthesis was described by Rawlings et al. [65], with its key step being the reductive alkylation of DNJ, which, in turn, was obtained as described below. Against SARS-CoV-2, *N*-nonyl-DNJ and NAP-DNJ were more active than MON-DNJ, but also with higher cytotoxicity [14].

### 3.2. N-Alkyl Derivatives Synthesized in Recent Years

In this section, we summarize the latest *N*-alkyl derivatives of DNJ studied for their antiviral activities (Table 2).

#### 3.2.1. 5′-Tocopheroxypentyl-DNJ (ToP-DNJ, **1**)

In 2018, Kiappes et al. [52] reported the synthesis of a new *N*-alkyl derivative of DNJ exhibiting antiflaviviral activity. The authors selected a form of vitamin E, specifically D-α-tocopherol, to conjugate to DNJ. The resulting compound, 5′-tocopheroxypentyl-DNJ (**1**, ToP-DNJ), showed a remarkable selectivity for GluII, both for isolated enzymes and in a whole cell system, which has not been reported for any other DNJ derivative. Moreover, it was hypothesized that the non-toxic moiety of vitamin E would be useful for tissue-targeting through accumulation in two body compartments of interest for antiviral therapy. In fact, d-α-tocopherol, after being absorbed in the gut, is stored in the liver, which is the target organ of HCV and a potential reservoir for DENV; it also accumulates in the membranes of immune cells, which are among the target cell types of DENV. Biodistribution studies carried out in Top-DNJ-treated mice demonstrated that, for both oral and intravenous administration routes, the highest amounts were detected in the liver. This liver-targeting property is attributed to its α-tocopherol moiety, too, which naturally directs it to the liver upon absorption. In addition, the IC_50_ of Top-DNJ for DENV inhibition in MDMΦs was reported to be 12.7 μM. The conjugation with vitamin E also improved plasma and liver half-lives. The synthesis of Top-DNJ was reported by Kiappes et al. [52]. It starts from d-α-tocopherol and makes use of tetra-*O*-acetyl DNJ, rather than DNJ as it is.

#### 3.2.2. 2-(3,4-Dihydroxyphenyl)-7-hydroxy-5-((10-((2*R*,3*R*,4*R*,5*S*)-3,4,5-trihydroxy-2-(hydroxymethyl)piperidin-1-yl)decyl)oxy)-4*H*-chromen-4-one (DNJ-20, **2**)

In 2025, Liu et al. [94] reported a study on a series of Traditional Chinese Medicine theory-inspired DNJ-flavonoid conjugates as α-glucosidase inhibitors. Interestingly, the flavonoid derivative **2** (DNJ-20) demonstrated high inhibition activity against α-glucosidase (α-Glu I), with an IC_50_ value of 55.3 μg/mL and also potent and broad-spectrum anti-coronaviral activity against SARS-CoV-2 pseudovirus (PsV), Delta and Omicron BA.5 variants, HCoV-229E and HCoV-OC43, with EC_50_ values up to 1.49 μM, which is more potent than UV-4. In addition, it had no observable cytotoxicity in HeLa-ACE2, HEK-293T, and Beas-2B cells.

#### 3.2.3. *N*-8′-(2′′-Tetrahydrofuranyl)octyldeoxynojirimycin (2THO-DNJ or UV-12, **3**) and (*N*-(8′-Ethoxyoctyl)deoxynojirimycin) (EOO-DNJ, **4**)

Perera et al. (2022) [60] studied the combination of GluI and GluII inhibition with higher iminosugar in DENV-infected dendritic cells: some DNJ-derivatives (**3** or 2THO-DNJ, and **4** or EOO-DNJ, given by Emergent BioSolutions Ltd. and Oxford Glycobiology Institute, respectively) and the previously studied *N*-nonyl-DNJ. The three compounds inhibited DENV secretion in a dose-dependent manner. ER GluI inhibition, at concentrations of 3.16 μM, was observed for the three compounds, along with a reduction in the specific infectivity of virions that were still secreted, as well as a reduction in DENV-induced tumor necrosis factor alpha secretion. The authors suggested that, beyond the reduction in viral secretion achieved with GluII inhibition, the iminosugar-mediated ER GluI inhibition may give rise to further benefits during DENV infection. The antiviral efficacy of these compounds against HAZV, responsible for Crimean–Congo hemorrhagic fever virus (CCHFV), is under study by using the surrogate Hazara Virus (HAZV) [95].

#### 3.2.4. UV-5 like DNJ-Valiolamine Derivatives (EB-0128, **5**; EB-0442, **6**; EB-0450, **7**; and EB-0686, **8**)

Karade et al. (2023) [64] reported several DNJ-valiolamine derivatives (UV-4-like and UV-5-like) as ER α-glucosidase I inhibitors for SARS-CoV-2 with EC_50_ values in the order of the submicromolar (0.42–1.61 μM), indicating that these agents were 2- to 8-fold more potent than UV-4B. The most potent belongs to the series of UV-5-like congeners, and was compound **5** (EB-0128).

#### 3.2.5. (2*R*,3*R*,4*R*,5*S*)-1–(5-(Adamantan-1-ylmethoxy)pentyl)-2-(hydroxymethyl)piperidine-3,4,5-triol (**9**) and (2*R*,3*R*,4*R*,5*S*)-1–(5-(Bicyclo[1.1.1]pentan-1-yl)pentyl)-3,4,5-trihydroxy-2-(hydroxymethyl)piperidin-1-ium Chloride (**10**)

Ferjancic et al. (2024) [89] synthesized and studied several derivatives of DNJ and evaluated their activity against SARS-CoV-2. Compounds **9** and **10** were potent anti-SARS-CoV-2 agents. Compound **9**, containing the adamantane moiety, had been previously reported [90] as a selective inhibitor of non-lysosomal glucosylceramidases (including β-glucosidase 2) and was regarded as inactive towards ER glucosidases involved in N-glycan trimming [96]. Compound **9** is the most potent iminosugar-based anti-SARS-CoV-2 agent reported thus far [90]. The two compounds act by a host-directed mechanism; thus, they should be more resilient to drug resistance.

#### 3.2.6. (3-(*tert*-Butyl)-1-cyclohexyl-1-(6-((2*R*,3*R*,4*R*,5*S*)-3,4,5-trihydroxy-2-(hydroxymethyl)piperidin-1-yl)hexyl)urea (IHVR-19029 or BSBI-1902, **11**); (2*R*,3*R*,4*R*,5*S*)-1-(6-(2,5-Difluorophenoxy)hexyl)-2-(hydroxymethyl)piperidine-3,4,5-triol, phenylether DNJ (IHVR-11029, **12**) and *N*-Cyclohexyl-*N*-(6-((2*R*,3*R*,4*R*,5*S*)-3,4,5-trihydroxy-2-(hydroxymethyl)piperidin-1-yl)hexyl)pivalamide, Pivalamide DNJ (IHVR-17028, **13**)

In 2013, Chang et al. [91] reported a study on three small molecules as derivatives of a previously reported compound, CM-10-18 as antiviral agents against representative viruses from viral families causing hemorrhagic fever, specifically EBOV, MARV, BVDV, DENV, and TCRV. The three compounds (IHVR-19029, BSBI-19029, **11**; IHVR-11029, **12;** and IHVR-17028, **13**) showed antiviral activity and significantly reduced the mortality of two of the most pathogenic hemorrhagic fever viruses, EBOV and MARV, in mice when administered via the intraperitoneal injection route. These compounds also showed a favorable adsorption, distribution, metabolism, and elimination (ADME) profile in comparison to the parent compound, as they did not significantly inhibit the activity of a panel of representative cytochrome P450 enzymes at 10 μM concentration. However, compounds **11** and **13** have low oral bioavailability, partly due to poor absorption, as shown by a high efflux ratio in the Caco-2 permeability experiment. Compound **11**, acting as both GluI and GluII inhibitors, was chosen as a representative compound. In 2017, Ma et al. (2017) [92] reported a study on a prodrug of compound **11**, specifically the tetrabutyrate analog. This prodrug demonstrated better in vivo pharmacokinetic properties in mice upon both oral and intravenous administration. Moreover, in vitro evaluation evidenced that the bioconversion of the prodrugs depends on the species: specifically, in mice, the prodrug was converted to **11** in the plasma and liver, while in humans, the conversion occurred mainly in the liver. In a successive paper by the same group, the association of **11** with favipiravir was also studied for Yellow fever and EBOV, with interesting results [97]. More recently, Reyes et al. (2021) [93] reported a study on a compound named ureido-*N*-hexyl deoxynojirimycin or BSBI-19029, which is likely compound **11**, that exhibits high activity against SARS-CoV-2 in A549-ACE2 cells (EC_90_ = 4 µM). Yesudhas et al. (2021) [98] reported that it has undergone clinical studies for SARS-CoV-2. The activities of compounds **11**–**13** have been recently summarized and analyzed for their potential in oncology, infectious diseases, and metabolic disorders [7].

## 4. Synthesis of Deoxynojirimycin and Its Derivatives

**Figure 2:**
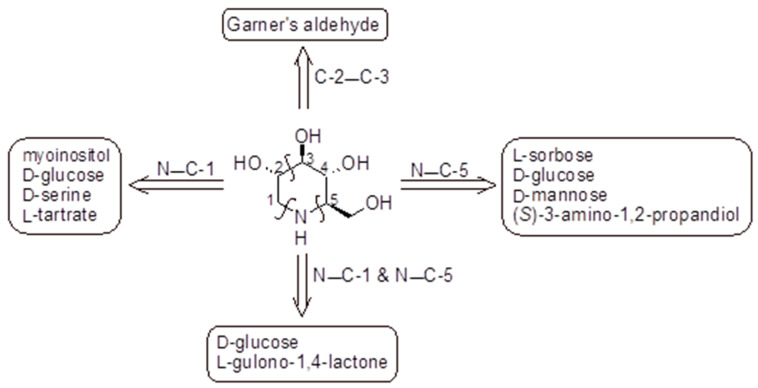
Preparation of DNJ based on the chiral pool approach: retrosynthetic analysis. The key steps closing the piperidine ring are highlighted above the arrows indicating the corresponding chiral starting materials [99,100,104].

The polyhydroxylated piperidine alkaloid DNJ may be considered as a key intermediate in the synthesis of its *N*-alkyl derivatives. The active isomer of DNJ is the (+)-DNJ, one out of the possible 16 stereoisomers, and its chemical synthesis is quite challenging due to the needed stereochemical control. The synthetic routes described in the literature and summarized below lead to this isomer (Figure 2). Most of them have been reviewed in several excellent papers [99,100], while more recent works were reviewed by Dahiya et al. (2025) [26]; they mostly rely on the chiral pool approach, including cheap starting materials. In Figure 2 a schematic retrosynthetic analysis is reported to highlight the main disconnections and corresponding starting materials. A relatively low number of elegant asymmetric syntheses were also reported and used either organocatalysis or enzymatic procedures [99,100,101,102]. A couple of preparations involved an optical resolution step [103]. Generally, the preparation of DNJ required numerous steps (from four to more than 10) and orthogonal protecting strategies, with the main involved reactions being reductive amination, intramolecular nucleophilic substitution reactions, ring closing metathesis, Huisgen azide alkene cycloaddition, or transamidification [99]. In most cases, DNJ was obtained in low to moderate overall yields (2–61%) with the most efficient methods starting from chirons and involving some chemoenzymatic steps [99].

A convenient large-scale synthesis of (+)-DNJ hydrochloride from D-glucuronolactone was reported by Best et al. (2010) [105] in an overall yield of up to 72%. Bagal et al. (2010) proposed the synthesis of (+)-DNJ obtained by resolution of (R*S*)-*N*(1)-benzyl-3-hydroxy-4-benzyloxy-2,3,4,7-tetrahydro-1H-azepine [106]. Iftikhar et al. (2017) [107] reported an improvement in the synthesis of the key step in the synthesis of DNJ, which is the preparation of 2,3,4,6-tetra-*O*-benzyl-D-glucono-δ-lactam, by using PCC as an oxidant, obtaining DNJ in 85% yield [108]. Alkylation of (+)-DNJ with suitable alkylating reagents gave most of the reported *N*-alkyl derivatives.

## 5. Mechanisms of Action Proposed for the GluII Inhibition of Iminosugars

Caputo et al. (2016) [88] examined the crystal structures of GluII in complex with DNJ, evidencing that the endocyclic nitrogen atom of DNJ interacts with D564 and occupies the −1 subsite. This interaction mimics the natural substrate (glucose), effectively inhibiting the enzyme reaction by preventing further catalytic activity. A unique structural feature in ER GluII, the F307 residue in the exclusion loop, confers specificity to these derivatives, reducing off-target effects. The alkyl chain of *N*-alkyl iminosugars stretches toward the side chains of the conserved residues F307 and F571, suggesting that these compounds act by blocking access to the +1 subsite as well as occupying the −1 subsite with the iminosugar ring. The butyl tail of NB-DNJ displaces the side chain of W525 in the +1 subsite, causing disorder and allowing interactions with residues like F307 and F571. In the case of MON-DNJ, the alkyl chain of the iminosugar is in two main conformations, with the major conformer docking against the exclusion loop F307 and the minor one docking against the hydrophobic side chain of F571, therefore reaching toward the +2 subsite. The longer chain also induces significant conformational changes in the α523–528 loop, making it unstable and enhancing inhibition. The mechanism of antiviral activity of NB-DNJ and NN-DNJ in BVDV and HIV models was investigated by Durantel et al. (2001) [45], who evidenced that the long-alkyl-chain compounds induced a viral envelope glycoprotein homodimer accumulation in the ER. In cells treated with these compounds, the overall quantity of E2 protein increased, which is most noticeable in E2-E2 homodimers. The functionalization of the alkyl chain, as in the DNJ-tocopherol conjugate ToP-DNJ (**1**), showed selective binding to ER GluII over other glucosidases, including those found in the gut [52]. Its high specificity for ER GluII is attributed to the aromatic tocopherol moiety, which interacts with a hydrophobic exclusion loop near the enzyme’s active site [52].

## 6. Considerations on Toxicity

Mehta et al. (2002) [41] analyzed the toxicity of fourteen *N*-alkyl derivatives of DNJ, evidencing that *N*-7-oxadecyl-DNJ and *N*-9′-methoxynonyl-1-deoxynojirimycin (MON-DNJ) were less toxic than *N*-nonyl-DNJ, as evaluated by MTT assay. In the study by Warfield et al. (2017) [44] against EBOV, the toxicity of several *N*-alkylated derivatives of DNJ was compared. NAP-DNJ was less cytotoxic (CC_50_ = 350 μM) than NN-DNJ, but more than MON-DNJ. NAP-DNJ, along with MON-DNJ, was selected to be further studied against EBOV and MARV in vivo, but in vivo efficacy in rodents and nonhuman primates was not demonstrated, also in this case. Thus, along with the length of the alkyl chain, lipophilicity must also be considered. The optimal compromise between antiviral efficacy and acceptable cytotoxicity of *N*-alkyl-DNJ derivatives was a side chain length of 8–9 carbons, and a Log*P* of approximately 2.8–3.0 [14]. To sum up, in these studies, MON-DNJ (UV-4) was found to be the least cytotoxic, followed by NAP-DNJ (UV-5) and *N*-oxadecyl-DNJ (UV-3). The compound with the highest toxicity was *N*-nonyl-DNJ (UV-2). It should be considered that, given the host-targeted mechanism of action of the iminosugars, some cytotoxic effects may occur. Recent compounds have been designed to overcome toxicity, such as the inhibition of the intestinal glucosidases in ToP-DNJ (**1**), which was designed to minimize the risk of gastrointestinal side effects of the antiviral iminosugar clinical candidates. Moreover, viral escape or adaptive mechanisms can occur, which may influence long-term therapeutic utility. DNJ and *N*-alkyl DNJ derivatives inhibit ER a-glucosidases, preventing glucose trimming and interaction with calnexin/calreticulin (CNX/CRT), thus representing a therapeutic target in infections by enveloped viruses that require interaction with CNX/CRT for the folding of functional glycoproteins. If folding is incomplete, the glycoprotein is reglucosylated, enabling cyclical interaction with chaperones until the native conformation is achieved, while persistently misfolded proteins enter the ER-associated degradation pathway for proteasomal degradation. However, the detection of triglucosylated viral glycoproteins in the presence of iminosugars, as in the case of studies with some influenza virus strains, demonstrates a pathway whereby partially or misfolded glycoproteins can be produced. In addition, some iminosugars can enhance the secretion of high-mannose glycoproteins, indicating that ER quality control may be bypassed by another enzyme, the Golgi-located endo-a-d-mannosidase, which cleaves the bond between glucose residues and the polymannose chain of the oligosaccharide. Therefore, iminosugar inhibition of oligosaccharide processing by ER Glu could be bypassed by endo-α-d-mannosidase [109].

## 7. Conclusions

Vaccination alone no longer guarantees global protection against viral threats, such as measles in Canada and North America, and the known COVID-19 pandemic. We can conclude with a sentence taken from Dwek et al. (2026) [110]: “If vaccines falter, broad-spectrum antivirals provide a global shield”. Antivirals offer a crucial complementary approach. However, virus-specific drugs require long times and high costs, often with minimal success, as seen at the beginning of the COVID-19 pandemic. Enveloped viruses hijack the protein production machinery of their host to replicate, and their replication/infectivity relies on the correct folding of their surface glycoproteins. This dependence on glycoprotein quality control for the viral replication cycle makes the host ERQC mechanisms an attractive target for the development of host-targeting antiviral agents, which may show broad-spectrum activity and a high genetic barrier to resistance. ER GluI removes the terminal glucose residue of N-linked glycans attached to nascent glycoproteins, enabling them to become substrates of ER Glu II, the main enzyme of the ERQC pathway. The key ERQC protein is GluII, which mediates quality control of the folding of nascent glycoproteins by trimming glucose residues from its substrate glycan Glc1-2Man9GlcNAc2. Broad-spectrum antivirals, such as host-targeting iminosugars, were suggested as a promising alternative [111]. Iminosugars mimic the glucose residue of the native substrate, causing the build-up of misfolded protein in the ER and preventing the secretion of correctly folded glycoproteins. In the case of enveloped virus infection, the inhibition of GluII can inhibit virion secretion or reduce infectivity of secreted virions and has been shown to elicit antiviral effects against a range of enveloped viruses in vitro and in vivo. Among GluII inhibitors, iminosugars derived from DNJ have revealed interesting activity as antivirals. Several compounds belonging to this class have been studied. However, their names generally create a lot of confusion among readers, since different names are used in the literature to refer to a single compound, and we have tried to address this confusion. In summary, the most interesting *N*-alkyl DNJ derivatives as antivirals in terms of efficacy and low toxicity are represented by MON-DNJ (UV-4) and its hydrochloride salt (UV-4B), which is already in clinical studies, and NAP-DNJ (UV-5). *N*-nonyl-DNJ (UV-2), although with high antiviral activity, is more toxic than the others. Generally, lengthening the alkyl chain determines an improvement in the antiviral activity. However, the introduction of heteroatoms should be taken into consideration, as it is often in favor of the antiviral activity, despite a shorter alkyl chain. In clinical studies, UV-4B exhibited a good safety profile up to a concentration of 1000 mg after administration as an oral solution formulation and demonstrated efficacy against measles and respiratory syncytial virus, highlighting its potential as a pan-respiratory antiviral targeting viruses most likely to cause pandemics. Generally, efficacy increases with alkyl chain length. Regarding toxicity, the alkyl chain length, along with lipophilicity, must be conceivably considered. The introduction of an oxygen atom generally decreases toxicity, to a greater or lesser extent, depending on its position. Log*P* values of about 2.8–3.0 seem to be the optimal compromise for obtaining antiviral efficacy and acceptable cytotoxicity. It must be considered that since viruses use cell glycosylation machinery, and therefore cell enzymes, such as ER GluI and GluII, and CNX/CRT chaperons for proper glycoprotein maturation, the interference with cell glycoprotein processing of infected cells remains a critical issue. Trying to limit the effects to the infected cells would be the goal, minimizing side effects, as gastrointestinal. In fact, an important limit of *N*-alkyl-DNJ derivatives is represented by gastrointestinal toxicity. The ideal antiviral drugs should be selective inhibitors of ER Glu, targeting allosteric sites unique to ER Glu and not substrate analogs targeting the catalytic center that may be similar among different glucosidases (including gut glucosidases). Among the *N*-alkyl derivatives of DNJ, the DNJ-tocopherol conjugate, ToP-DNJ, in which DNJ is chemically linked to α-tocopherol, a form of vitamin E that is absorbed through the gut and directed to the liver, holds promise as an antiviral agent, given its enzyme and organ selectivity and low gastrointestinal side-effects. Finally, newly studied *N*-alkyl derivatives of DNJ are analyzed at the end of the paper, highlighting that research in this field still represents a challenge.

## 8. Future Perspectives

Despite promising in vitro and in vivo data, several critical gaps remain before GluII inhibitors can be convincingly translated into clinical practice. A comprehensive toxicity profile, such as chronic exposure and organ-specific safety, is still missing and will be crucial for long-term use. It could be interesting to further explore whether *N*-alkyl DNJ iminosugars are preferable to other host-targeted antiviral strategies in terms of efficacy, safety, and resistance potential. Moreover, in vivo quantification of the therapeutic window and pharmacokinetics of the most active analogs has not yet been clearly established. Finally, to define the tissue distribution, precise pharmacokinetic/pharmacodynamic characterization is needed, as well as metabolic stability and target interaction studies, also in order to distinguish on-target from off-target effects, particularly towards non-ER glucosidases. An approach could be using orally available prodrugs that are hydrolyzed in the circulation and intracellular compartments by ubiquitous esterases, thus potentially avoiding inhibition of gut glucosidases. In addition, the prodrug approach could increase the time of the drug in plasma to achieve an efficacious dose and improve the metabolic stability of the drug to increase the half-life. The prodrug of IHVR-19029 (**11**) as a tetrabutyrate analog is under study. On the other hand, the improvement in selectivity of DNJ derivatives towards ER Glu, with increased hydrophobicity and ER delivery and distribution, could be another strategy. MON-DNJ was suggested as a new frontier in antiviral pharmacology, offering a paradigm shift that could transform global responses and prevent the catastrophic losses seen during the COVID-19 pandemic. The use of combination therapy of iminosugars with other drugs to enhance antiviral activity could represent a promising strategy for advancing next-generation iminosugars as antivirals. Finally, our consideration on nomenclature issues: a standardized nomenclature should be adopted in future studies, at least when salts of the same compound are concerned. The use of the acronym UV-4B, which refers to the hydrochloride salt of UV-4, is misleading since it might indicate that UV-4B and UV-4 are different compounds, which is not completely true. UV-4B might be given as UV-4 HCl or UV-4 hydrochloride.

## Figures and Tables

**Table 1:** Antiviral activities of the most common *N*-alkyl derivatives of DNJ.

Compound	Name	Biological Activity	Ref.
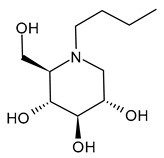	*N*-butyl DNJ (NB-DNJ or UV-1 or AT2221 or miglustat)	IC_50_ = 125–200 μM (BVDV) CC_50_ > 5000 μM (MDBK cells) IC_50_ = 100–500 μM (HBV) CC_50_ > 5000 μM (HepG2 2.2.15 cells)	[41]
		IC_50_ = 6.00 ± 7.31 µM (DENV-2-infected MDMΦs) CC_50_ = 24.903 ± 10.506 µM (MDMΦs)	[56]
		IC_50_ = 162 μM (DENV) CC_50_ > 500 μM (Vero cells)	[57]
		EC_50_ = 10.6 μM (DENV-infected MDMΦs)	[43]
		IC_50_ = 32.95 μM (EBOV) IC_50_ = 47.72 μM (MARV) CC_50_ > 500 μM (Vero cells)	[44]
		EC_50_ = 41 ± 22 µM (Vero E6 infected with SARS-CoV-2) CC_50_ > 1000 µM (Vero cells) EC_50_ = 80.5 ± 23 µM (Calu-3 infected with SARS-CoV-2) CC_50_ > 1000 µM (Calu-3 cells)	[58]
		IC_50_ = 170 µM (SARS-CoV-2) CC_50_ > 1000 µM (Calu-3 cells)	[31]
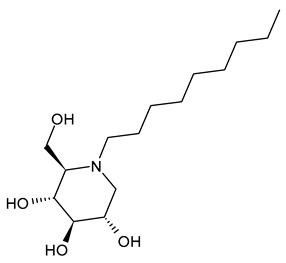	*N*-nonyl-DNJ (NN-DNJ or UV-2)	IC_50_ = 6 μM (BVDV) CC_50_ = 175 μM (MDBK cells) IC_50_ = 1–10 μM (HBV) CC_50_ = 175 μM (HepG2 2.2.15 cells)	[41]
		EC_50_ = 4.0 ± 0.5 μM (Huh7.5 infected with HCV) IC_50_ = 0.54 ± 0.08 (GluI) CC_50_ = 87 ± 8.7 μM (Huh7.5 cells)	[48]
		IC_50_ = 0.91 ± 0.4 µM (DENV-2 infected MDMΦs) CC_50_ = 317 µM (MDMΦs)	[57]
		IC_50_ = 9 μM (DENV) CC_50_ = 125 μM (Vero cells)	[56]
		EC_50_ = 1.25 μM (DENV-infected MDMΦs)	[43]
		IC_50_ = 15.22 μM (EBOV) IC_50_ = 28.66 μM (MARV) CC_50_ = 125 μM (Vero cells)	[44]
		IC_50_ ~ 6.84 μM (HPV E5 viroporin)	[59]
		IC_50_ = 3.3 ± 1.5 μM (DENV-2-infected imDCs) CC_50_ = 479 ± 211 µM (imDCs)	[60]
		IC_50_ = 0.4 ± 0.2 µM (H3N2-infected MDCK) IC_50_ = 1.9 ± 0.8 µM (H1N1-infected MDCK)	[61]
		IC_50_ = 4.63 µM (SARS-CoV-2 ENG2/20) CC_50_ > 500.0 μM (Calu-3 cells)	[31]
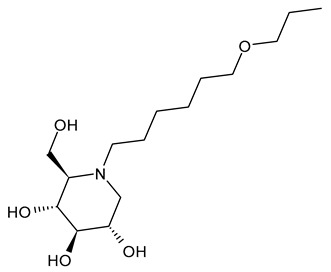	*N*-7-oxadecyl-DNJ (UV-3)	IC_50_ = 30 μM (BVDV) CC_50_ > 2000 μM (MDBK cells) IC_50_ = 100 μM (HBV) CC_50_ > 2000 μM (HepG2 2.2.15 cells)	[41]
		IC_50_ = 41 μM (DENV) CC_50_ > 500 μM (Vero cells)	[57]
		IC_50_ = 34.98 μM (EBOV) IC_50_ = 47.72 μM (MARV) CC_50_ > 500 μM (Vero cells)	[44]
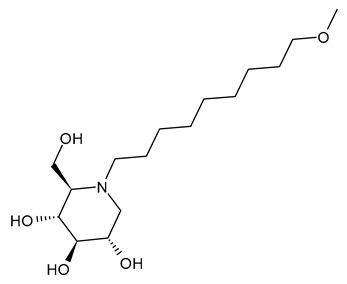	*N*-9-methoxy-nonyl-DNJ (MON-DNJ or UV-4)	IC_50_ = 3.0 μM (BVDV) CC_50_ > 2000 μM (MDBK cells) IC_50_ = 3.0 μM (HBV) CC_50_ > 2000 μM (HepG2 2.2.15 cells)	[41]
		IC_50_ = 3.09 ± 3.93 μM (DENV-2-infected MDMΦs) CC_50_ = 3.150 ± 1.211 μM (MDMΦs)	[56]
		IC_50_ = 17 μM (DENV) CC_50_ > 500 μM (Vero cells)	[57]
		IC_50_ = 29.97 μM (EBOV) IC_50_ = 47.72 μM (MARV) CC_50_ > 500 μM (Vero cells)	[44]
		IC_50_ = 51.7 µM (SARS-CoV-2 ENG2/20) IC_50_ = 14.1 µM (SARS-CoV-2 Omicron B.1.1.529) IC_50_ = 21.3 µM (HCoV OC43) IC_50_ = 0.5371 µM (GluI) CC_50_ > 1000 μM (Calu-3 and HuH-7 cells)	[31]
UV-4 HCl	UV-4B	IC_50_ = 2.10 µM (DENV-1 SH29177) IC_50_ = 6.49 µM (DENV-2 NGC) IC_50_ = 3.64 µM (DENV-3 SL 5-29-04) IC_50_ = 2.78 µM (DENV-4 H241) CC_50_ > 1 mM (Vero cells) IC_50_ = 0.16 µM (Mouse ER GluI)	[62]
		EC_50_ = 2.694 µM (SARS-CoV-2-infected ACE2-A549 cells) EC_50_ = 2.489 µM (SARS-CoV-2-infected Caco-2 cells) EC_50_ = 4.369 µM (SARS-CoV-2 beta variant-infected ACE2-A549 cells) EC_50_ = 6.816 µM (SARS-CoV-2 beta variant-infected Caco-2 cells) CC_50_ > 400 μM (ACE2-A549 and Caco-2 cells)	[63]
		EC_50_ = 3.32 µM (SARS-CoV-2) CC_50_ > 100 μM (ACE2-A549 cells)	[64]
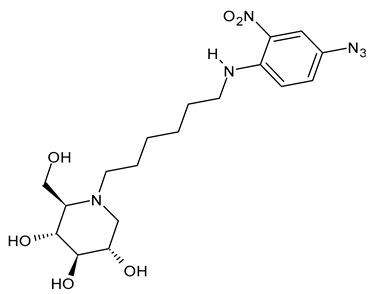	*N*-[*N*-(4-azido-2-nitrophenyl)-6-aminohexyl]-DNJ (NAP-DNJ or UV-5)	IC_50_ = 2 μM (DENV) CC_50_ = 350 μM (Vero cells)	[57]
		IC_50_ = 7.859 μM (EBOV) IC_50_ = 6.359 μM (MARV) CC_50_ = 350 μM (Vero cells)	[44]
		IC_50_ = 0.017 ± 0.001 (purified rat liver GluI)	[65]
		IC_50_ = 9.92 µM (SARS-CoV-2 ENG2/20) CC_50_ = 132 μM (Calu-3 cells)	[31]
		EC_50_ = 0.58 µM (SARS-CoV-2) CC_50_ > 100 μM (ACE2-A549 cells)	[64]

**Table 2:** Antiviral activities of the latest *N*-alkyl derivatives of DNJ.

Structure	Compound	Antiviral Activity	Ref.
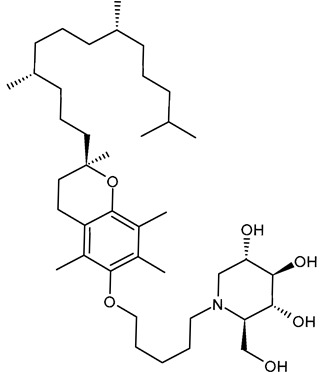	**1** ToP-DNJ	IC_50_ = 12.7 μM (DENV-infected MDMΦs)	[52]
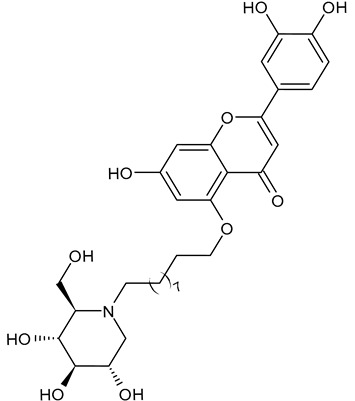	**2** DNJ-20	EC_50_ = 5.13 µM (PsV entry) EC_50_ = 7.52 µM (PsV packaging)	[88]
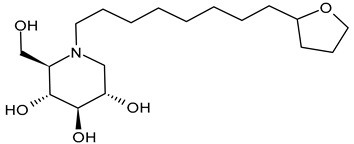	**3** 2THO-DNJ or UV-12	IC_50_ = 1.6 ± 0.8 μM (DENV-2 infected imDCs) CC_50_ = 443 µM (imDCs)	[60]
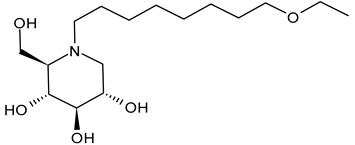	**4** EOO-DNJ	IC_50_ = 3.1 ± 1.3 μM (DENV-2 infected imDCs) CC_50_ > 1000 µM (imDCs)	[60]
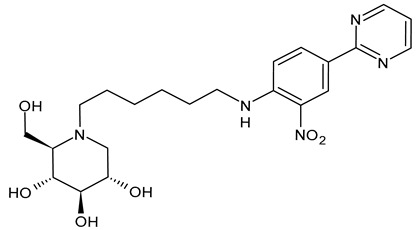	**5** EB-0128	EC_50_ = 0.42 µM (SARS-CoV-2) CC_50_ > 100 μM (ACE2-A549 cells)	[64]
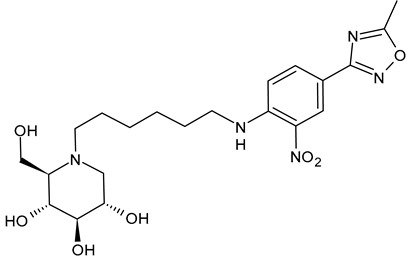	**6** EB-0442	EC_50_ = 0.44 µM (SARS-CoV-2) CC_50_ > 100 μM (ACE2-A549 cells)	[64]
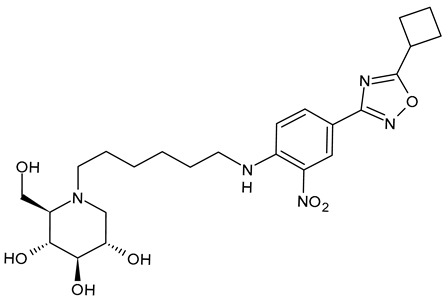	**7** EB-0450	EC_50_ = 0.72 µM (SARS-CoV-2) CC_50_ = 40.74 μM (ACE2-A549 cells)	[64]
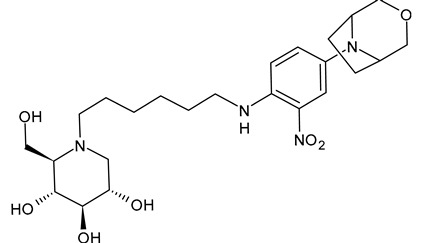	**8** EB-0686	EC_50_ = 1.61 µM (SARS-CoV-2) CC_50_ > 100 μM (ACE2-A549 cells)	[64]
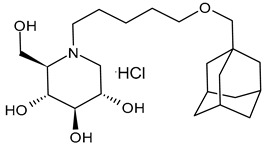	**9**	EC_90_ = 1.94 µM (SARS-CoV-2 Omicron BA.1 strain-infected ACE2-A549 cells)	[89]
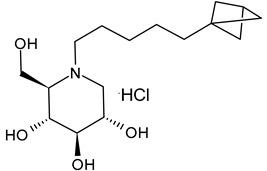	**10**	EC_90_ = 3.83 µM (SARS-CoV-2 Omicron BA.1 strain-infected ACE2-A549 cells)	[90]
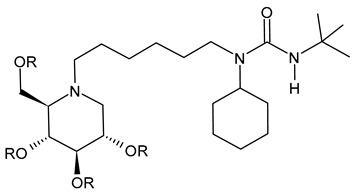	**11** IHVR-19029 or BSBI-19029 (R = H) **11 Tetrabutyrate** IHVR-19029 or BSBI-19029 tetrabutyrate (R = CH_3_CH_2_CH_2_CO)	EC_50_ = 0.25 ± 0.05 µM (BVDV) EC_90_ = 16.3 ± 7.8 µM (BVDV) CC_50_ > 500 µM (MDBK cells) EC_50_ = 0.74 ± 0.3 µM (TCRV) EC_90_ = 52.5 ± 38.9 µM (TCRV) CC_50_ > 500 µM (Huh7.5 cells) EC_50_ = 1.25 ± 1.1 µM (DENV) EC_90_ = 22.5 ± 10.6 µM (DENV) CC_50_ > 500 µM (BHK cells) EC_90_ = 4 µM (SARS-CoV-2 in A549-ACE2 cells)	[91,92,93]
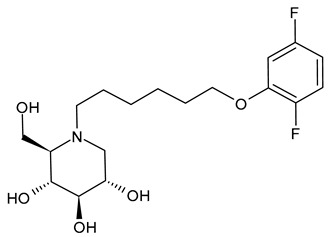	**12** IHVR-11029	EC_50_ = 1.3 ± 0.7 µM (BVDV) EC_90_ = 16 ± 7.9 µM (BVDV) CC_50_ > 500 µM (MDBK cells) EC_50_ = 3.3 ± 2.7 µM (TCRV) EC_90_ = 69 ± 37.7 µM (TCRV) CC_50_ > 500 µM (Huh7.5 cells) EC_50_ = 0.75 ± 0.06 µM (DENV) EC_90_ = 6.3 ± 3.5 µM (DENV) CC_50_ > 500 µM (BHK cells)	[7,91]
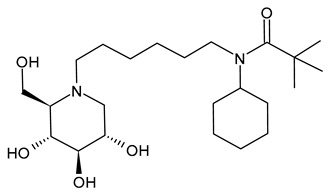	**13** IHVR-17028	EC_50_ = 0.4 ± 0.6 µM (BVDV) EC_90_ = 16 ± 12µM (BVDV) CC_50_ > 500 µM (MDBK cells) EC_50_ = 0.26 ± 0.08 µM (TCRV) EC_90_ = 26.7 ± 20.9 µM (TCRV) CC_50_ > 500 µM (Huh7.5 cells) EC_50_ = 0.3 ± 0.03 µM (DENV) EC_90_ = 1.7 ± 0.8 µM (DENV) CC_50_ > 500 µM (BHK cells)	[7,91]

## Data Availability

No new data were created or analyzed in this study. Data sharing is not applicable to this article.

## Abbreviations

The following abbreviations are used in this manuscript:

BVDV	Bovine viral diarrhea virus
CC_50_	Half maximum cell cytotoxicity
CCHFV	Crimean–Congo hemorrhagic fever virus
CNX	Calnexin
COVID-19	Coronavirus Disease 2019
CRT	Calreticulin
DENV	Dengue virus
DENV-2	Dengue virus serotype 2
DNJ	Deoxynojirimycin
EC_50_	Half maximal effective concentration
EBOV	Ebola virus
ER	Endoplasmic reticulum
ERQC	Endoplasmic reticulum quality control
GluI	α-Glucosidase I
GluII	α-Glucosidase II
HAZV	Hazara virus
HBV	Hepatitis B virus
HCV	Hepatitis C virus
HIV	Human immunodeficiency virus
HPV	Human papillomavirus
IC_50_	Half maximal inhibitory concentration
MARV	Monocyte-derived macrophages
PsV	SARS-CoV-2 pseudovirus
SARS-CoV-1	Severe acute respiratory syndrome coronavirus 1
SARS-CoV-2	Severe acute respiratory syndrome coronavirus 2
TCRV	Tacaribe virus
VSV	Vesicular stomatitis virus
WNV	West Nile virus
